# Effect of high-intensity versus low-intensity praziquantel treatment on HIV disease progression in HIV and *Schistosoma mansoni *co-infected patients: a randomised controlled trial

**DOI:** 10.12688/wellcomeopenres.14683.2

**Published:** 2019-04-03

**Authors:** Andrew Abaasa, Gershim Asiki, Andrew Obuku Ekii, Josephine Wanyenze, Pietro Pala, Govert J. van Dam, Paul L.A.M. Corstjens, Peter Hughes, Song Ding, Giuseppe Pantaleo, Pontiano Kaleebu, Alison M. Elliott, Anatoli Kamali

**Affiliations:** 1 London School of Hygiene & Tropical Medicine, London, Keppel Street, WC1E 7HT, UK; 2 MRC UVRI & LSHTM Uganda Research Unit, Entebbe, Uganda; 3 Leiden University Medical Center, Leiden, The Netherlands; 4 Centre Hospitalier Universitaire Vaudois, Rue du Bugnon, 46, 1011 Lausanne, Switzerland

**Keywords:** HIV, Schistosoma, co-infection, high-intensity praziquantel treatment, disease progression

## Abstract

**Background: **It has been hypothesised that *Schistosoma* co-infection exacerbates HIV progression, and hence anthelminthic intervention in co-infected individuals will delay it. We evaluated effects of high-intensity versus low-intensity praziquantel treatment of schistosomiasis on HIV disease progression among co-infected patients from fishing populations around Lake Victoria, Uganda.

**Methods**: Between August 2012 and September 2015, we conducted an open-label randomised, controlled trial. Adults, antiretroviral therapy-naïve, CD4 counts ≥350 cells/μl, HIV and *S. mansoni *co-infected, were randomised 1:1 to praziquantel (40mg/kg) given quarterly (starting at enrolment) or annually (starting 12 weeks after enrolment; such that low-intensity participants were still untreated when sampled at 12 weeks). A non-randomised HIV-positive *S. mansoni-*negative comparison group was recruited. The primary outcome was mean change in plasma viral load at 12 and 60 weeks.

**Results:** In total 363 participants (high-intensity 113, low-intensity 113, comparison group 137) were recruited; 96 (85.0%), 97 (85.8%) and 107 (78.1%) completed 60 weeks of follow up, respectively. Adjusting for baseline age and viral load, the geometric mean ratio (aGMR [95%CI]) viral load for high-intensity vs low-intensity groups at 12 weeks was 0.90 [0.65, 1.25] p=0.55 and at 60 weeks 1.88 [0.78, 4.53] p=0.16. Results in the comparison group were similar to trial arms. High-intensity, compared to low-intensity, treatment resulted in substantially lower *S. mansoni* prevalence at all follow up visits (p<0.05).

**Conclusions:** In communities with a high burden of both *S. mansoni *and HIV infection, high-intensity treatment of *S. mansoni *does not delay HIV progression despite relevant benefit for parasite clearance.

**Trial registration:** ISRCTN15371662 (17/11/2016)

## Introduction

HIV and helminth co-infections are common in resource constrained settings. Globally, an estimated 25% of HIV-positive individuals are reported to be co-infected ^1– 4^. In Africa this figure is estimated at 50% ^5^. Some studies have suggested that helminth co-infection could lead to faster HIV progression ^6– 9^. If this is true, interventions that treat helminths could help to avert HIV disease progression among co-infected people.

An observational study conducted in Ethiopia among HIV-positive individuals co-infected with *Ascaris* or *Trichuris* showed a decrease in HIV plasma viral load after treatment of helminths with albendazole ^10^; however, a systematic review that included a randomised trial and four observational studies found that evidence regarding the benefit of anti-helminth therapy for HIV viral load, CD4 count, and clinical progression was inconclusive ^11^. Subsequently, in Entebbe, Uganda, we found that treating pregnant women with albendazole resulted in a modest decrease in HIV load ^12^. With the exception of the large “HEAT” trial by Walson and colleagues ^13^, available studies to date have been limited in design by small sample sizes, short duration of follow-up and lack of attention to the possibility of species-specific effects ^14^. The HEAT trial found no benefit of routine quarterly albendazole and annual praziquantel for HIV progression. However, helminth infection status was investigated only at the end of the trial at which point the prevalence of *Schistosoma* in the control arm (having received no routine anthelminthic treatment) was modest; thus, the HEAT trial offers little insight into the specific, potential benefits of treating schistosomiasis ^13^.

Effects of *Schistosoma mansoni* deserve special attention: this is a systemic infection with strong immunomodulatory effects. These regulatory effects are required for the long-term survival of adult worms within the host ^15^, but inflammation against egg antigens is also required in order for eggs to migrate from mesenteric blood vessels, through the tissues and into the intestinal lumen ^16^. Interactions between helminths and HIV, mediated by immunological mechanisms, may therefore be especially important for schistosomiasis. In addition, HIV- *Schistosoma* co-infection is particularly common among fishing communities, such as those on the shores and islands of Lake Victoria, where *S. mansoni* infection is almost universal ^17^ and HIV prevalence among adults is up to 37% ^18^. These are recognised key populations with respect to HIV infection and regarded as likely reservoirs for the continuing HIV epidemic in the general population. Therefore, any impact of *S. mansoni* co-infection on HIV replication could have far-reaching consequences. 

A prospective study in Kenya with a variable duration of follow up found no benefit of treatment of *S. mansoni* on HIV load ^19^. In Uganda, we observed a transient increase in viral load following treatment with praziquantel ^20^. However, neither we, nor our colleagues in Kenya, included an untreated control group in these initial studies and thus the impact of praziquantel treatment on HIV progression remained unknown. Kallestrup and colleagues, in Zimbabwe, included a comparison group and found that, at three months, individuals treated for schistosomiasis (predominantly *S. haematobium*) had a smaller increase in viral load than individuals who had not been treated ^21, 22^.

Given these inconsistent results, we sought to evaluate the effect of high-intensity (quarterly) treatment in comparison with low-intensity (annual) praziquantel treatment on HIV disease progression, in a large, well-powered study, among patients co-infected with HIV and *S. mansoni* from fishing populations around Lake Victoria, Uganda. This was aimed at assessing possible benefits of more frequent anthelminthic treatment among hard-to-reach populations whose access to anti-retroviral treatment is limited.

## Methods

### Trial registration

This trial was registered with the International Standard Registered Clinical/Social Study Number (ISRCTN) registry on the 17/11/2016. Trial number: ISRCTN15371662. A completed CONSORT checklist is available as Supplementary File 1

***Study design.*** This was an open label randomised controlled trial. HIV-positive adults were recruited. *Schistosoma mansoni* infected study participants were randomised to high-intensity versus low-intensity praziquantel treatment in the ratio of 1:1. The high-intensity treatment group received immediate treatment with two doses of praziquantel (40mg/kg) one week apart followed by praziquantel at 12 weeks, and then every 12 weeks. The low-intensity treatment group received a single dose of praziquantel (40mg/kg) annually (in keeping with standard Uganda government policy) the first treatment being delayed to 12 weeks from enrolment in order to determine the short-term effects of treatment by comparison with an untreated group and to replicate the Zimbabwe study ^22^. In parallel, we recruited a comparison group of HIV-positive individuals with no detectable *S. mansoni* infection. Initially it was planned that the comparison group would not receive any praziquantel treatment; later the protocol was amended such that participants in this group received praziquantel at 12 weeks to conform with standard of care in fishing communities. All participants received albendazole 400mg at weeks 12, 36 and 60 in keeping with policy for the control of nematode infections. Participants were followed for 60 weeks. All treatments were directly observed. 

***Outcomes.*** The primary study outcome was log _10_ plasma HIV-1 RNA level at 12 and 60 weeks of follow up. Secondary outcomes were CD4 counts, clinical progression of HIV (defined by clinical events such as opportunistic infections, and WHO staging) and mortality; and reduction of *S. mansoni* infection prevalence and intensity. Immunological investigations in this cohort will be reported separately.

***Study setting.*** The study was conducted in fishing communities on the shores of Lake Victoria in Masaka district, Uganda, where HIV prevalence among adults was estimated to be 29% and *S. mansoni* infection more than 50% ^18, 23, 24^.

***Inclusion and exclusion criteria.*** Inclusion criteria were age at least 18 years, HIV and *S. mansoni* co-infection, antiretroviral therapy (ART) naïve, not in advanced HIV WHO stage III or Stage IV, CD4 T cell count >350cells/mm ^3^ (i.e. not eligible for ART initiation according to prevailing guidelines at the time of the study); willing and consenting to provide laboratory specimens for stool tests, HIV viral loads, CD4 count, full blood count; available for follow up for 15 months and willing to provide locator information for tracking purposes. Participants were excluded from the study if they met any one of the following criteria: women pregnant or planning to be pregnant; had taken praziquantel in the preceding three months; had symptomatic helminth infection (Hb <8g/dl, bloody diarrhoea, clinically apparent liver disease (vomiting blood, hepatosplenomegaly)); had high-intensity of S *. mansoni* infection (egg count >2000 eggs/g; these received immediate praziquantel treatment). Enrolment to the comparison group followed similar criteria except that participants had to be *S. mansoni* negative on analysis of three stool samples by microscopy. 

***Study procedures and measurements.*** *Screening visits:* Trained field workers mobilised the targeted population through house to house HIV counselling and testing. Those found to be HIV-infected were referred to the study clinic at Lambu fish landing site, which is the largest fishing village in the study area located about 50km from Masaka town. After written informed consent, they were requested to provide three stool samples on consecutive days to ascertain *S. mansoni* infection status. During the screening visit, blood samples were also taken for CD4 count and urine from women for pregnancy testing, to complete the eligibility assessment. Volunteers were then encouraged to return within 2 weeks for enrolment.

*Randomisation (enrolment visit):* During this visit, individuals who met the study criteria were enrolled and baseline clinical history and examination including WHO HIV staging were conducted (Baseline questionnaire available as Supplementary File 2). Blood samples were collected for plasma viral load levels and CD4 counts. Eligible participants were randomly allocated to one of the two treatment groups (high-intensity or low-intensity praziquantel treatment) using random permuted blocks of variable size by an independent statistician. A randomisation list containing study numbers with the allocated treatment codes was provided to the study team and participants who were eligible were assigned the next available number until the required sample size was reached. Participants in the high-intensity group received the first praziquantel dose (directly observed) during the enrolment visit, while treatment was deferred for those in the low-intensity group to the 12 weeks’ visit. At each visit, treatment was given after blood and stool samples had been collected. Neither participants nor investigators were masked as to treatment allocation.

Similar processes were followed for the comparison group except that these participants were *S.mansoni* negative on all three stool samples.

*Allocation concealment:* The study statistician generated two randomisation lists: i) containing trial numbers from which eligible participants were sequentially allocated the next available number ii) containing trial numbers and the allocation arms. List (i) was held at the trial clinic, while list (ii) was held by the trial statistician. When an eligible participant was recruited and allocated the next available number on the randomisation list (i), the statistician was contacted to provide the treatment allocation arm on list (ii).

*Follow-up visits:* From the enrolment visit, participants were scheduled to return every 12 weeks until their exit. Participants in the high-intensity group made an additional visit one week after enrolment to receive their second dose of praziquantel. At every follow-up visit, clinical evaluation, urine pregnancy testing (women), praziquantel administration for those in the high-intensity group and plasma storage were undertaken. CD4/CD8 counts, *S. mansoni* infection (single stool tests and circulating anodic antigen (CAA)) were conducted every 3 months starting from enrolment day. Plasma viral load assessments were done at enrolment, week 12 and week 60.

## Laboratory analysis

*Stool analysis:* Each stool sample was processed and evaluated using the Kato-Katz technique ^25^. Two slides were made from each sample, each using a template designed to capture 41.7mg of stool. Slides were examined within 30 minutes of preparation for hookworm eggs, and the following day for other ova, including *S. mansoni.* The presence of other helminth eggs was recorded and the burden of infection based on the number of eggs per gram of stool calculated according to WHO criteria ^26, 27^.

*Blood samples:* Serological testing for HIV-1 was performed using Alere determine ^™^ rapid test HIV1/2,Cat/ref7D2343 Abbott, Japan, with all positive tests confirmed by Statpack (HIV1/2STAT-PAK DIPSTICK Cat/refHIV303 Inverness, USA) with Unigold (Trinity Biotech Uni-Gold HIV Cat/ref120652, Ireland) as tie-breaker (the prevailing Uganda Ministry of Health algorithm at the time of the study). The CD4 lymphocyte count was determined using Multiset ^™^ software DR-DOS 5.0 system, V1.4 on a FACSCalibur machine (Becton Dickinson, USA). Plasma HIV-1 RNA was quantified using the Ampliprep/Taqman V2.0 kit Cat number; 05212294190, Roche Molecular systems Inc, Pleasanton, USA HIV-1 viral load assay, which has been shown to quantify the subtypes of HIV-1 prevalent in Uganda and had a detection level of 20 copies of viral RNA/mL. Serum CAA was assessed, after all samples had been collected, to define *S. mansoni* infection status and intensity more precisely: Plasma CAA was measured using the up-converting phosphor lateral flow assay in three sets; (set 1) >50pg/ml was considered positive, 20–50pg/ml indecisive and <20pg/ml negative, (set 2) >30pg/ml was considered positive, 10–30pg/ml indecisive and <10pg/ml negative and (set 3) >30pg/ml was considered positive, 13–30pg/ml indecisive and <15pg/ml negative ^23^. All Laboratory investigations were performed at MRC/UVRI and LSHTM Uganda Research Unit clinical diagnostics laboratory.

## Ethical considerations

The study was approved by Uganda Virus Research Institute (UVRI) Research Ethics Committee (REC), GC/127/12/02/01 and Uganda National Council for Science and Technology (UNCST), HS1141. To address challenges of delayed treatment among those randomised to the low-intensity group, in relation to direct, helminth-induced pathology, we excluded people who were symptomatic, or with a high egg burden (>2000epg), and likely to benefit from immediate treatment. When participants became eligible for ART (according to the prevailing Uganda Ministry of Health guidelines) they were immediately referred to a local ART provider.

## Role of the funding sources

The research leading to these results was funded primarily from the European Community's Seventh Framework Programme (FP7/2007–2013) under EC-GA n° 241642. As well, the research was supported by the UK Medical Research Council (MRC) and the UK Department for International Development (DFID) under the MRC/DFID Concordat agreement. AME was supported by a Wellcome Trust senior fellowship, grant number 095778. The funders did not have access to the data and were not involved in the analysis or interpretation of the results and did not provide input regarding the decision to publish this manuscript.

## Statistical methods

*Sample size estimation* was based on evaluation of the primary outcome: viral load measured at study exit among participants treated with high-intensity, compared to those on low-intensity, praziquantel. We aimed to recruit and follow to completion 188 HIV and *S. mansoni* co-infected participants, giving approximately 89% power to detect as significant a difference in log _10_ viral load copies/mL at 60 weeks of 0.35 log _10_ copies/mL. These assumptions were based on the baseline viral load in the rural community cohort in Uganda (unpublished) and a within group standard deviation in the log _10_ copies/mL of 0.75. Due to the anticipated loss to follow up of 20% (estimated from the 18 months’ fisher folk cohort) ^28^ the overall sample size was increased to 226 participants (113 per group).

*Data handling and analysis:* Data were double-entered and verified in Microsoft Access 2003 (Microsoft Corporation, Redmond, WA) and analysed using Stata 14 (Stata Corp, College Station, Texas, USA). Participant baseline socio-demographics and clinical characteristics were summarised using counts and percentages, by study group, for categorical variables and means and standard deviation (SD) for continuous variables. The analysis was by intention to treat (ITT). The prevalence of *S. mansoni* and other helminth infections, and egg counts (transformed on natural logarithm), were compared between the study groups using Chi-square tests and geometric means respectively. The viral loads showed skewed distributions, with a number of results (61-overall (12 at baseline)) as undetectable. An offset from zero of 10 copies/mL was added to all the viral loads, to allow suitable logarithmic analysis. Results were transformed to log _10_ (viral loads) and analysed by linear regression using bootstrapping with 10,000 iterations. Regression coefficients and confidence limits were back-transformed to express results as ratios of geometric means. All the primary analyses were adjusted for baseline age and viral loads and included all participants to the end of follow up, regardless of whether or not they initiated antiretroviral treatment. Similar approaches were followed for CD4 counts though the transformation was on natural logarithm and no corrections were made. A Kaplan Meier curve with log-rank test was used to compare the clinical course of HIV disease (WHO staging) between the study groups. Mortality between the groups was compared by proportions.

Two sensitivity analyses were performed: one, for the trial analysis, excluding viral loads and CD4 count results of the participants that initiated ART during the trial and those with baseline undetectable viral loads; the other for analyses of the comparison group, excluding individuals found to have *S. mansoni* infection at any time point. The exclusion in the latter first considered all with indecisive CAA results (10–30pg) as negative and secondly as positive. Similar approaches as above were followed.

## Results

**Figure 1.**
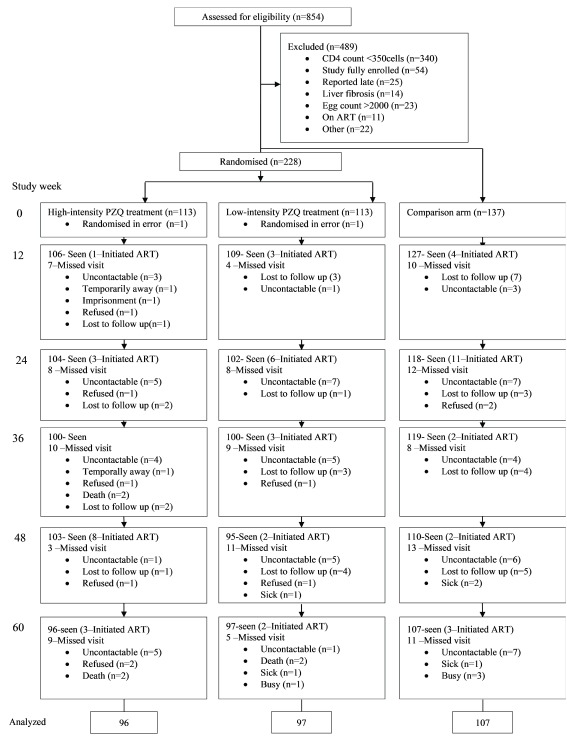
CONSORT flow diagram. PZQ - praziquantel.

*Study profile:* Between August 2012 and September 2015, a total of 854 participants were screened and 363 (42.7 %) enrolled (113 in each of the high-intensity and low-intensity groups and 137 in the comparison group). The most common reason for exclusion was CD4 count <350 cells ( Figure 1). We also excluded two participants, one in each trial group, that were randomised in error. A total of 36 participants were lost during the trial; loss was similar between the trial groups. Fifty-three (15 high-intensity, 16 low-intensity and 22 comparison group) participants initiated antiretroviral treatment during follow up, on average 3 participants per visit.

*Participant characteristics at baseline:* Participants’ baseline characteristics are presented in Table 1. The characteristics were similar in the two trial groups except that participants in the high-intensity group were slightly younger, a smaller proportion was single (never married), and the prevalence of other helminths (Hookworm, *Ascaris*, and *Trichuris*) was lower than in the low-intensity group. The comparison group had a higher proportion of women compared to the trial groups, reflecting the lower prevalence of *S. mansoni* infection among women than men in these communities. The baseline CD4 count and viral loads were comparable in all the study groups.

**Table 1.** Baseline information by randomisation group and non-randomised comparison group.

		Number (%)		
Characteristic	Category	High-intensity PZQ (n=113)	Low-intensity PZQ (n=113)	Comparison (n=137)
Sex	Male	87(77.0)	90(79.7)	72(52.6)
	Female	26(23.0)	23(20.3)	65(47.4)
Age (years)	Median (IQR)	29 (24-33)	30 (26-36)	30 (26-36)
Age group	18–24	29(25.7)	21(18.6)	25(18.3)
	25–34	64(56.6)	55(48.7)	62(45.2)
	35–59	20(17.7)	37(32.7)	50(36.5)
Education	None	11(9.7)	10(8.9)	12(8.8)
	Primary	88(77.9)	86(76.1)	102(75.4)
	Secondary	14(12.4)	17(15.0)	23(16.8)
Marital	Single, never married	11(9.7)	20(17.7)	14(10.2)
	Married	62(54.9)	60(53.1)	82(59.8)
	Single, ever married	40(35.4)	33(29.2)	41(30.0)
Occupation	Fishing/related	88(77.9)	83(73.4)	83(73.4)
	Small scale business	9(8.0)	8(7.1)	8(7.1)
	Bar/restaurant	4(3.5)	7(6.2)	7(6.2)
	Other	12 (10.6)	15(13.3)	15(13.3)
CD4 count	Mean ln (SD)	6.5(0.33)	6.4(0.31)	6.4(0.36)
†Viral Load	Mean log _10_ (SD)	4.5(1.01)	4.5(0.74)	4.4(0.98)
Schistosoma (Kato Katz microscopy)	Prevalence	113(100)	113(100)	0(0.0)
	*Geometric mean egg count (95%CI)	244.2(192.3-310.1)	228.0(181.6-286.3)	N/A
Schistosoma (serum circulating anodic antigen (CAA))	Prevalence	99(87.6)	101(89.4)	47(34.3)
	*Geometric mean concentration pg/ml (95%CI)	1708.2(1178.6- 2475.6)	1877.8(1277.3- 2760.7)	482.2(293.3- 792.7)
Other worms	Prevalence	11(9.7)	21(18.6)	22(16.1)

PZQ praziquantel. †12 volunteers (9-Low-intensity PZQ arm and 3-comparison) had undetectable viral loads at baseline. * Geometric mean among those infected. Figures in brackets are percentages unless otherwise indicated in column 2, IQR-Interquartile range.

A total of 300 (82.6%,) participants completed the study follow up at 60 weeks and had the primary outcome determined. Study completion did not differ by the trial arm, standard 97 (85.8%), intensive 96 (85.0%) and comparison 107 (78.1%) p=0.202. A higher proportion of females (24.6%) did not complete the study follow up compared to 14.1% of the males p=0.014, but otherwise completers and non-completers were similar in regards to other baseline characteristics.

*HIV viral load:* The primary objective was to compare the effect of high-intensity versus low-intensity treatment with praziquantel on HIV disease progression by comparing viral loads between baseline and 12 weeks, and between baseline and 60 weeks in the two study groups. There was no statistical evidence of difference in mean log _10_ viral loads between the high-intensity and low-intensity groups at 12 weeks, p=0.55 ( Table 2). After adjusting for baseline age group and viral load, the geometric mean ratio (aGMR) for high-intensity vs low-intensity treatment was 0.90; 95%CI (0.65, 1.25), p=0.55. There was a slightly higher mean log _10_ viral load in the high-intensity group compared to low-intensity group at 60 weeks: after adjusting for baseline age group and viral load, the aGMR was 1.88; 95%CI (0.78, 4.53), p=0.16. Excluding those with undetectable viral load at baseline, and those that initiated ART during follow up, there was no evidence of a difference in viral load at 60 weeks between the high-intensity and low-intensity treatment groups (aGMR 1.01 95%CI (0.64, 1.95), p=0.71).

**Table 2.** Adjusted ratio of geometric means for the primary outcome (viral load) and CD4 counts at 12 and 60 weeks by randomisation and comparison group.

Outcome	Randomisation Group	12 weeks			60 weeks		
		Mean (SD) ^$^	aGMR *	P-value	Mean (SD) ^$^	aGMR *	P-value
Viral load	Low-intensity PZQ	4.2 (1.16)	1.00		3.6 (1.57)	1.00	
	High-intensity PZQ	4.3 (1.08)	0.90 (0.65-1.25)	0.55	4.0 (1.22)	1.88 (0.78-4.53)	0.16
	Comparison	4.1 (1.22)	1.18 (0.83-1.69)	0.36	3.6 (1.47)	0.92 (0.39-2.18)	0.84
CD4 count	Low-intensity PZQ	6.3 (0.38)	1.00		6.3 (0.40)	1.00	
	High-intensity PZQ	6.4 (0.38)	0.99 (0.93-1.07)	0.96	6.3 (0.40)	0.94 (0.86-1.02)	0.15
	Comparison	6.3 (0.41)	0.99 (0.93-1.07)	0.97	6.2 (0.42)	1.00(0.91-1.09	0.98

PZQ praziquantel. *aGMR - adjusted ratio of geometric means, ^$^ mean –Viral load transformed on log10 and CD4 on natural logarithm, adjusted for age and baseline viral load or CD4 count; “Low-intensity PZQ” was the reference group

The comparison group had patterns of viral load change similar to the low-intensity group.

*CD4 count:* There were no significant differences in mean CD4 count between the study groups at any time during follow up, even after adjusting for baseline age group and CD4 count; high-intensity vs low-intensity at 60 weeks aGMR 0.94 (0.86, 1.02), p=0.15 ( Table 2). The comparison group did not differ from either trial group.

*Schistosoma mansoni and other helminth infections:* The prevalence of *S. mansoni* as assessed by microscopy was substantially lower in the high-intensity treatment group compared to the low-intensity group at 12 weeks (21.9% vs 72.5% (p<0.01); as expected, given that the low-intensity group was still untreated at this time) and at 60 weeks (6.6% vs 32.3% (p<0.01). Corresponding reductions in geometric mean egg counts among those infected were observed ( Table 3). Although the prevalence of other helminths was somewhat higher in the high-intensity group at baseline, it did not differ significantly between the two study groups during follow up ( Table 3). The prevalence of *S. mansoni* as assessed by CAA was also substantially lower in the high-intensity treatment group compared to the low-intensity at 12 weeks and 60 weeks: 74.2% vs 94.9% (p<0.01) and 29.2% vs 73.4% (p<0.01) respectively.

**Table 3.** Schistosoma prevalence and geometric mean egg count by study week and randomisation and comparison groups.

Time point	Prevalence geometric mean)	High-intensity PZQ (n=113)	Low-intensity PZQ (n=113)	p-value ^^^	Comparison n=137	p-value ^$^
12 weeks						
Kato Katz microscopy	Prevalence	23/105 (21.9%)	79/109 (72.5%)	<0.01	9/124 (7.3%)	<0.01
	*Geometric mean egg count (95%CI)	115.7 (73.9-181.1)	288.4 (215.8-385.6)	<0.01	82.3 (32.8-206.4)	0.43
CAA	prevalence	66/97 (68.0%)	86/97 (88.7%)	<0.01	13/82 (15.9%)	<0.01
	*Geometric mean pg CAA / mL (95% CI)	369.1 (247.2-551.2)	2041.5 (1395.5-2986.7)	<0.01	219.5 (100.6-478.8)	0.44
36 weeks						
Kato Katz microscopy	Prevalence	9/98 (9.2%)	22/99 (22.2%)	0.01	11/119 (9.2%)	0.99
	*Geometric mean egg count (95%CI)	61.3 (33.1-113.6)	136.1 (90.2-205.3)	0.05	38.7 (27.6-54.3)	0.17
60 weeks						
Kato Katz microscopy	Prevalence	6/91 (6.6%)	31/96 (32.3%)	<0.01	8/107 (7.5%)	0.81
	*Geometric mean egg count (95%CI)	54.6 (22.4-133.0)	191.5 (124.5-294.7)	0.01	59.1 (36.4-95.9)	0.55
CAA	prevalence	26/89 (29.2%)	69/94 (73.4%)	<0.01	14/103 (13.6%)	0.01
	*Geometric mean pg CAA / mL (95%CI)	295.5 (152.0- 574.4)	695.1 (463.8-1041.6)	0.03	103.3 (62.8-169.9)	0.04
Other helminths						
12 weeks	Prevalence	6/106 (5.7%)	13/109 (11.9%)	0.11	9/124 (7.3%)	0.63
36 weeks	Prevalence	7/98 (7.1%)	7/99 (7.1%)	1.00	5/119 (4.2%)	0.37
60 weeks	Prevalence	1/91 (1%)	1/96 (1%)	0.99	0/109 (0.0%)	0.60

PZQ praziquantel. * Geometric mean among those infected, ^High-intensity PZQ to Low-intensity PZQ group, ^$^ High-intensity PZQ vs comparison, CAA-serum circulating anodic antigen

Although the comparison group had no evidence of *S. mansoni* infection by microscopy at baseline, infection was detected by CAA in 41.5%; in line with this result, a small proportion of comparison group participants were positive by microscopy and by CAA during follow up (CAA-positive 23.2% and 13.6% at 12 and 60 weeks, respectively; Table 3). When members of the comparison group with *S. mansoni* infection detectable by either method were excluded from the analysis of viral load, viral load measurements in this group were still similar to the low-intensity group: aGMR 0.94 (0.77–1.15), p=0.55 and 0.90 (0.54–1.49), p=0.68 at 12 and 60 weeks (all indecisive CAA results considered as negative). Similar results were obtained when indecisive CAA results were considered as positive (date not shown).

**Figure 2.**
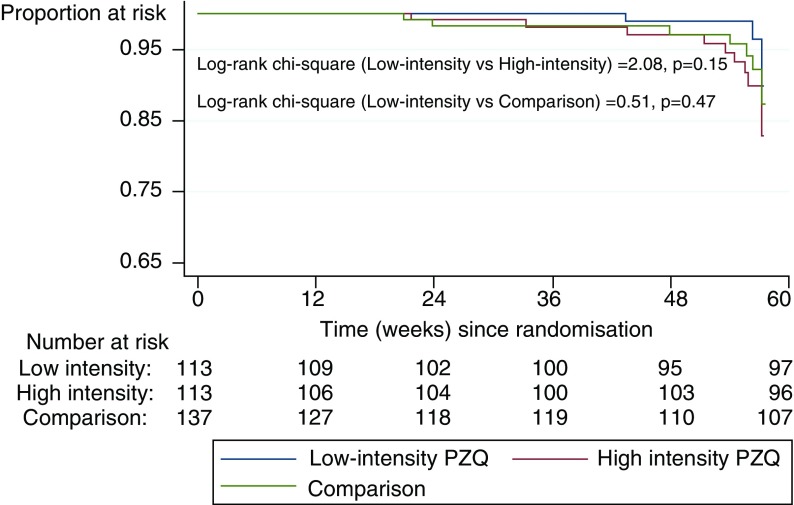
Volunteers moving up in WHO staging by study group. PZQ – praziquantel.

*Mortality and progression to AIDS:* In total six participants (4-high-intensity and 2-low-intensity group) died during follow up. Twenty-five participants (10-high intensity, 5- low intensity and 10-comparison arm) progressed in WHO clinical staging during follow up. Based on WHO clinical staging, progression to AIDS was more likely to occur in high-intensity treatment and comparison groups compared to the low-intensity group, although this finding was not statistically significant (log-rank chi-square (low-intensity vs high-intensity) 2.08, p=0.15; and log-rank chi-square (low-intensity vs comparison group) 0.51, p=0.47 ( Figure 2)). 

## Discussion

This randomised clinical trial was designed to establish whether high-intensity treatment of *S. mansoni* with praziquantel delays HIV disease progression. We used HIV viral load, CD4 count and clinical parameters as markers of disease progression. We found no benefit of praziquantel treatment of *S. mansoni* for HIV disease progression. If anything, at week 60 of follow up, HIV viral loads were slightly higher among participants who received high-intensity treatment than among those who received low-intensity treatment. In addition, analysis of outcomes in the comparison group indicated that *S.mansoni* infection *per se*, under either treatment regimen, had no effect on HIV disease progression.

Our study was not blinded, but it is unlikely that the low-intensity group received praziquantel outside the trial protocol since it is not widely available in the community clinics and pharmacies; the infection prevalence (based on microscopy) and CAA concentration at 12 weeks (i.e. prior to the first treatment in this group) remained high in this group. A marked difference in *S. mansoni* prevalence, as assessed by Kato Katz microscopy, emerged between the low-intensity and high-intensity treatment groups by 12 weeks, and persisted during follow up. The more sensitive CAA analysis showed that complete clearance of infection was slower than it appeared using microscopy of single stool samples, and this could have obscured a true effect of eliminating *S. mansoni* during the early part of follow up; however, a substantial difference had been achieved by 60 weeks. Follow up Kato Katz and CAA analyses in the comparison group indicated that some members were, in fact, *S. mansoni* infected but a sensitivity analysis restricting the comparison group to individuals negative on all tests still showed no evidence of statistical difference relative to the trial low-intensity group.

In this study, there was a hint of an adverse effect of treating schistosomiasis on HIV load – the aGMR indicated a higher viral load in the high-intensity treatment arm, although the confidence interval was wide (aGMR 1.88 (95%CI 0.78–4.53). This is in agreement with earlier observations in Kisumu, Kenya ^19^ and in Uganda ^20, 29^. The cohort study in Kisumu demonstrated a moderate rise in mean HIV-1 plasma viral load among patients who received praziquantel treatment, but the study lacked a comparison group. Similarly, we previously demonstrated a transient rise in plasma HIV viral load in a cohort of HIV- *S. mansoni* co-infected patients in Uganda, more marked among subjects with higher intensity *S. mansoni* infections ^20^. These prior studies were limited by short follow up periods and lack of treatment randomisation. In terms of mechanism, the factors producing the type 2 responses to worm antigens released following praziquantel treatment ^20^ may affect the extracellular environment and antigen presenting cells (APC)s that determine the functional fate of naïve T cells recognizing HIV antigens, priming a phenotype less effective in hindering HIV replication. Additionally, the activated, proliferating *S. mansoni* specific CD4 T cells responding to the circulating antigen surge might themselves constitute additional targets for HIV infection and replication, supporting a transient increase in viral load. 

Our findings contrast with the results of the earlier Zimbabwe study, the only similar randomised trial of praziquantel treatment to recruit individuals of confirmed *Schistosoma* infection status and to address HIV-related outcomes, which we sought to confirm. The Zimbabwe trial was a smaller study, with shorter follow up and lower power than this study. The Zimbabwe trial included participants with both *S. haematobium* and *S. mansoni*; infection intensity (at least for *S. mansoni*) was markedly lower than in our study (with mean egg counts of 3–4 epg of stool, compared to our geometric mean of >200 epg) ^21^. Differences in infection intensity as well the involved species may explain differences in impact on the immune system (and hence on HIV replication). Low-intensity infections are more likely to be readily cleared by a single dose of treatment.

Our study strengths included a prolonged follow up period, sufficient sample size and randomisation of treatment. The results provide strong evidence that, in communities with a high burden of both *S. mansoni* and HIV infection, high-intensity treatment of *S. mansoni* does not delay HIV progression despite benefits for parasite clearance. Our study limitation included a challenge that fishing communities are predominantly males and they constituted about 75% of the study population in the two randomized groups. However, a subgroup analysis stratifying by gender, though underpowered still showed that high-intensity treatment of *S. mansoni* does not delay HIV progression in males as well as females. We therefore conclude that, unfortunately, treatment of *S. mansoni* is not likely to contribute to mitigating the HIV epidemic among fishing communities.

## Data availability

The MRC/UVRI and LSHTM Uganda Research Unit has a data sharing policy accessible through this link https://www.mrcuganda.org/publications/data-sharing-policy. The policy summarizes the conditions under which data collected by the MRC/UVRI and LSHTM Uganda Research Unit can be made available to other bona fide researchers, the way in which such researchers can apply to have access to the data and how data will be made available if an application for data sharing is approved. Should any of the other researchers need to have access to the data from which this manuscript was generated, we authors will make it available to them. The corresponding and other co-author emails have been provided and could be contacted anytime.

## Supplementary material

Supplementary File 1 – Completed CONSORT checklist

.

Supplementary File 2 – Baseline questionnaire
