# Supplementary material for: Effect of high-intensity versus low-intensity praziquantel treatment on HIV disease progression in HIV and Schistosoma mansoni co-infected patients: a randomised controlled trial
Source: Wellcome Open Res. 2019 Apr 3;3:81. Originally published 2018 Jul 5. [Version 2] doi: 10.12688/wellcomeopenres.14683.2 (PMC6241569; doi:10.12688/wellcomeopenres.14683.2)
Supplement: Supplementary file 2 [file wellcomeopenres-3-16601-s0001.tgz › c3a3adb9-44a1-4d21-be15-cefaea12d835.pdf]

IDNO |_S__|_I__|_S__|___|___|___|___|

SIS PROTOCOL Form 2: Clinical Assessment (CLA) - *Collect these data on every prospective volunteer at the enrolment and every follow up visit*

PART 1: PARTICIPANT IDENTIFICATION AND PHYSICAL MEASUREMENTS

QNo.	Questions	RESPONSE	**Codes**
101	SIS Trial number	|_ _|___|___|___|___|	**TRNO**
102	Date of visit	|__|__| |__|__|__| |_2_|0__|_1_|__| Day Month Year	**VISDAT**
103	Visit number	|___|___|. |___|	**VISN**
104	Measure temperature	|___|___|.|___| degrees centigrade	**TEMP**
105	Measure weight	|___|___|___|Kg (to nearest Kg)	**WT**
106	Measure height	|___|___|___|cm (only at enrolment)	HT
107	Blood Pressure<br>Pulse	Systolic |___|___|___|mmHg<br>Diastolic |___|___|___| mmHg<br>Pulse |___|___|___|/min	SYS<br>DIA<br>PULSE

IDNO |_S__|_I__|_S__|___|___|___|___|

PART 02: Medical history and Physical Examination

	**Questions**	RESPONSE	
201	Does the volunteer have any major health complaint today? ***(if no skip to 204)***	Yes 1<br>No 2	**PHEXAM**
202	***Record if symptoms or signs are present from each of these systems. (Enter 1 if present or 2 if absent in the box provided)***	***Write short and concise descriptions of symptoms and signs if present and duration, below***	
	General **[GEN]** |___|		**RGEN**
	ENT **[ENT]** |___|		**RENT**
	Cardiovascular **[CVS]** |___|		**RCVS**
	Pulmonary**[PULM]** |___|		**RPULM**
	Abdominal**[ABDOM]** |___|		**RABDOM**
	Pelvic Exam (if female) for male indicate N/A**[PELV]** |___|		**RPELV**
	Musculoskeletal**[MSS]** |___|		**RMSS**
	Skin **[SKIN]** |___|		**RSKIN**
	Genito-urinary **[GUT]** |___|		**RGUT**
	Other -Specify **[OTHER]** |___|		**ROTHER**
203	Diagnosis	______________________________	**DIAG**
204	Record any WHO staging clinical events volunteer had in the past (probe using the WHO staging form as a guide)		**PWHO**

205	Does the volunteer have any new HIV WHO clinical staging event?	Yes 1<br>No 2	WHOEV
206	If yes, record the new event		NEVENT
207	Current WHO clinical Stage	¦___¦	CWHO
208	Has any medication other than trial drug been prescribed to the participant today?	Yes 1<br>No 2	DRUG
209	If yes list the drug codes (maximum 4) ***Use drug code list***	¦___¦ ¦___¦ ¦___¦<br>¦___¦	CDRUG]
210	Has the patient started ART?	Yes 1<br>No 2	ART
211	If yes, specify the ART combination code and date of starting	¦___¦<br>|__|__| .|__|__|__| 201|_|	ARTCD<br>ARTDATE

	Code	(dd/mmm/yyyy)
Clinician		
Reviewed		
