# Supplementary material for: Effect of high-intensity versus low-intensity praziquantel treatment on HIV disease progression in HIV and Schistosoma mansoni co-infected patients: a randomised controlled trial
Source: Wellcome Open Res. 2019 Apr 3;3:81. Originally published 2018 Jul 5. [Version 2] doi: 10.12688/wellcomeopenres.14683.2 (PMC6241569; doi:10.12688/wellcomeopenres.14683.2)
Supplement: Peer review history

# Peer review: Reviewer response for version 2

**DOI:** 10.21956/wellcomeopenres.16601.r35215

**Published:** 2019-04-04

**Authors:** Judd L. Walson

**Affiliations:** 1 Departments of Global Health, Medicine (Infectious Disease), Pediatrics and Epidemiology, University of Washington, Seattle, WA, USA

The authors have adequately addressed the concerns raised in my prior review. Some of the limitations remain but the authors have done a very thorough job in responding clearly to each comment.

I have read this submission. I believe that I have an appropriate level of expertise to confirm that it is of an acceptable scientific standard.

# Peer review: Reviewer response for version 1

**DOI:** 10.21956/wellcomeopenres.15987.r34261

**Published:** 2018-11-15

**Authors:** Birgitte Jyding Vennervald

**Affiliations:** 1 Section for Parasitology and Aquatic Pathobiology (PAP), Faculty of Health and Medical Sciences, University of Copenhagen, Copenhagen, Denmark

This is a very interesting paper reporting the results of an open-label Randomised Controlled Trial (RCT) on the effect of intensive, 4 times per year vs. standard once yearly PZQ treatment on viral load among HIV and *Schistosoma mansoni *co-infected people living in fishing communities near lake Victoria. A non-randomised HIV-positive *S. mansoni-*negative comparison group was recruited as well.

 The primary study outcome was viral load at 12 and 60 weeks of follow up. Secondary outcomes were CD4 counts and the clinical progression of the HIV infection and reduction of *S. mansoni* infection prevalence and intensity.

 The results showed no statistically significant diffrences in viral load between the high-intensity vs low-intensity treatment groups at 12 weeks and 60 weeks and the overall conclusion of the paper is that in fishing communities with high *S. mansoni *and HIV infection prevalences, high-intensity treatment of S. mansoni does not delay HIV progression.

 The paper is clearly and well written. Overall the study is well conducted; the results described in a clear and concise manner and the conclusions drawn based on the results are sound and justified.

 I am very pleased to see that the authors report their results findings despite the fact that they found no significant differences between the two arms. This is highly important for the scientific community and generally for society that negative results are published. Well done. We should see more of that.

 I have some minor questions or comments to various sections of the paper:

1. The parasitological diagnosis was based on Kato-Katz technique with preparation of two slides per stool sample. I would have liked the authors to state the amount of stool used for a slide, since this may vary (25mg to 50 mg) and have an impact of the overall sensitivity of the method.
2. At the baseline examination and randomisation participants were providing three stool samples on three consecutive days. However, as far as I can see, parasitological results at follow-up time points are all based on a single stool. This has implications for the sensitivity of the test. Kato-Katz is not a very sensitive test and sensitivity is very low with just a single stool sample post treatment. This is why the CAA assay is very useful and important to include as an additional diagnostic measure. It would have been good to include these points in the discussion.
3. I do not really see the point in having the group of *S. mansoni *negatives. Parasitological and CAA analysis reveal that some of them are in fact infected at 12 weeks and they are treated with PZQ just like the low-intensity PZQ group. Furthermore, since they are *S. mansoni *negative or very slightly infected despite living in a high transmission fishing community means that they may differ from the two randomisation groups with respect to parameters, which have not been investigated but which may be of importance.
4. I am amazed to see that despite very intense PZQ treatment (every 12 weeks) it is not possible to bring *S. mansoni *infection down to almost nothing in this group of adults. *S. mansoni *is a tough parasite to treat. I am wondering if *Schistosoma haematobium *is easier and if this may play a role when comparing with the Zimbabwean *S. haematobium *study?
5. I do not quite understand figure 2 based on the figure legend. Maybe the legend could be expanded.
6. Could this maybe be re-phrased: “producing the type 2 bias of responses to worm antigens”. Maybe just remove “bias of”. Whenever I read it I stumble on type 2 bias and try to figure out how statistics come into this.

I have read this submission. I believe that I have an appropriate level of expertise to confirm that it is of an acceptable scientific standard.

# Response

**Published:** 2019-03-26

**Authors:** Andrew Abaasa

**Affiliations:** MRC/UVRI and LSHTM Uganda Research Unit, Uganda

**Effect of high-intensity versus low-intensity praziquantel treatment on HIV disease progression in HIV and *Schistosoma mansoni *co-infected patients: a randomised controlled trial**

****

**Abaasa et al**

****

**Response to the reviewer’s comments**

****

**Reviewer Two**

**Birgitte Jyding  Vennervald**, Section for Parasitology and Aquatic Pathobiology (PAP), Faculty of Health and Medical Sciences, University of Copenhagen, Copenhagen, Denmark 

 Approved

 This is a very interesting paper reporting the results of an open-label Randomised Controlled Trial (RCT) on the effect of intensive, 4 times per year vs. standard once yearly PZQ treatment on viral load among HIV and *Schistosoma mansoni *co-infected people living in fishing communities near lake Victoria. A non-randomised HIV-positive *S. mansoni-*negative comparison group was recruited as well.

 The primary study outcome was viral load at 12 and 60 weeks of follow up. Secondary outcomes were CD4 counts and the clinical progression of the HIV infection and reduction of *S. mansoni* infection prevalence and intensity.

 The results showed no statistically significant diffrences in viral load between the high-intensity vs low-intensity treatment groups at 12 weeks and 60 weeks and the overall conclusion of the paper is that in fishing communities with high *S. mansoni *and HIV infection prevalences, high-intensity treatment of S. mansoni does not delay HIV progression.

 The paper is clearly and well written. Overall the study is well conducted; the results described in a clear and concise manner and the conclusions drawn based on the results are sound and justified.

 I am very pleased to see that the authors report their results findings despite the fact that they found no significant differences between the two arms. This is highly important for the scientific community and generally for society that negative results are published. Well done. We should see more of that.

 I have some minor questions or comments to various sections of the paper:

 1.     The parasitological diagnosis was based on Kato-Katz technique with preparation of two slides per stool sample. I would have liked the authors to state the amount of stool used for a slide, since this may vary (25mg to 50 mg) and have an impact of the overall sensitivity of the method.

***Response: Thank you for this observation, the amount of the stool used for slides was 41.7 mg.  This has been included in the manuscript.***

 2.     At the baseline examination and randomisation participants were providing three stool samples on three consecutive days. However, as far as I can see, parasitological results at follow-up time points are all based on a single stool. This has implications for the sensitivity of the test. Kato-Katz is not a very sensitive test and sensitivity is very low with just a single stool sample post treatment. This is why the CAA assay is very useful and important to include as an additional diagnostic measure. It would have been good to include these points in the discussion.

***Response: In our second paragraph of the discussion section, we allude to this***.

 3.     I do not really see the point in having the group of *S. mansoni *negatives. Parasitological and CAA analysis reveal that some of them are in fact infected at 12 weeks and they are treated with PZQ just like the low-intensity PZQ group. Furthermore, since they are *S. mansoni *negative or very slightly infected despite living in a high transmission fishing community means that they may differ from the two randomisation groups with respect to parameters, which have not been investigated but which may be of importance.

***Response:  It is plausible that an infection such as schistosomiasis exacerbates HIV progression and that the effect is not reversible by treatment - because of persistence of helminth antigen, for example, and/or lasting impact on the immune system.  As indicated in the discussion, we consider the results from the comparison group useful to address this possibility – there was no substantial difference in progression between the comparison group and either treatment arm.  We do agree, however, that there were important (and largely inevitable) differences between the comparison group and the infected groups and we acknowledge that all comparisons with that group should be taken with caution and as secondary results. We draw our major findings from the two randomized arms.***

 4.     I am amazed to see that despite very intense PZQ treatment (every 12 weeks) it is not possible to bring *S. mansoni *infection down to almost nothing in this group of adults. *S. mansoni *is a tough parasite to treat. I am wondering if *Schistosoma haematobium *is easier and if this may play a role when comparing with the Zimbabwean *S. haematobium *study?

***Response: We thank the reviewer for this interesting comment.***

 5.     I do not quite understand figure 2 based on the figure legend. Maybe the legend could be expanded.

***Response: The legend has been expanded to align PZQ with high-intensity not to give an impression it is with the comparison arm.***

 6.     Could this maybe be re-phrased: “producing the type 2 bias of responses to worm antigens”. Maybe just remove “bias of”. Whenever I read it I stumble on type 2 bias and try to figure out how statistics come into this.

***Response:  “bias of” has been removed***

# Peer review: Reviewer response for version 1

**DOI:** 10.21956/wellcomeopenres.15987.r33727

**Published:** 2018-10-19

**Authors:** Paul Garner

**Affiliations:** 1 Liverpool School of Tropical Medicine (LSTM), Liverpool, UK

1. The authors examine the question of whether helminth infection-with Schistosomiasis-exacerbates HIV infection. The authors give PZ to S mansoni infected people, and those on the low intensity schedule were untreated at 12 weeks so a direct comparison of no treatment with PZ 40 dose was possible in terms of mean change in geometric mean of viral load. The treatment reduced S mansoni but did not show any impact on mean viral load change.
2. This is an important study and is highly relevant in a research question where there seem to be strong beliefs there is an effect, yet effects to date have been mixed but always quite small, and as such the evidence base is at risk of selective publication and selective reporting. The authors have therefore done a service to science in ensuring this study, which does not demonstrate an effect, has been published. They also appear to have adhered to the protocol and not sought secondary outcomes or subgroup analyses that risk generating spurious results.
3. It is a fascinating also because the authors were able to evaluate the effect of PZ and treating the infection in people with HIV that was not suppressed by ARVs. This was because it was conducted prior to the WHO recommendation to treat all people living with HIV with ARVs irrespective of CD counts. So this study is “as good as it will ever get” in testing whether treatment has any influence on HIV progression.
4. The study is well-written. The background explains studies to date and the gap in the literature leading to this study. Whilst this is a basic expectation of the background section, so many authors do not do this or do it badly, surprisingly; I will use this as an example of good practice in our teaching!  and the methods are clearly explained. The follow up is good and the results well presented.
5. It is appropriately reported without any attempt to overinterpret the results. Given the complexity of the study the authors have good numbers recruited. Very few people were started on ARVs during the course of the study and the sensitivity analysis showed this did not, by chance, influence the results, which might have happened if there was chance imbalance in the numbers treated in comparator groups.
6. The one point that would really help understand the context-and this is a broader concern with these studies of this kind around treating helminths in HIV (including the studies of albendazole used for soil transmitted helminth infection in people with HIV) is the ambiguity around the purpose of the study: is it truly believed by the authors that this could be potentially important component of treatment by delaying progression of the disease as stated in the first sentence of the discussion? Or that treatment of schisto could have some public health impact on transmission as outlined at the end of paragraph 3 in the introduction? Or is it simply a randomised explanatory trial to elucidate immune mechanisms with helminth infection? This is the only strongly recommended change I would want to see in the amended version.
7. With the absence of any demonstrable effect in this study, it seems most unlikely, given the dramatic effectiveness of ARVs in viral suppression, that further studies would be worthwhile. Indeed, given patients would need to be on ARVS, with such small putative possible effects of the treatment, the studies would have to be extremely large, probably so large that no-one would fund them. I think it would be extremely worthwhile reporting in their discussion. 

I have read this submission. I believe that I have an appropriate level of expertise to confirm that it is of an acceptable scientific standard.

# Response

**Published:** 2019-03-26

**Authors:** Andrew Abaasa

**Affiliations:** MRC/UVRI and LSHTM Uganda Research Unit, Uganda

**Effect of high-intensity versus low-intensity praziquantel treatment on HIV disease progression in HIV and *Schistosoma mansoni *co-infected patients: a randomised controlled trial**

****

**Abaasa et al**

****

**Response to the reviewer’s comments**

****

**Reviewer Three**

**Paul Garner**, Liverpool School of Tropical Medicine (LSTM), Liverpool, UK 

 Approved

 1.     The authors examine the question of whether helminth infection-with Schistosomiasis-exacerbates HIV infection. The authors give PZ to S mansoni infected people, and those on the low intensity schedule were untreated at 12 weeks so a direct comparison of no treatment with PZ 40 dose was possible in terms of mean change in geometric mean of viral load. The treatment reduced S mansoni but did not show any impact on mean viral load change.

 2.     This is an important study and is highly relevant in a research question where there seem to be strong beliefs there is an effect, yet effects to date have been mixed but always quite small, and as such the evidence base is at risk of selective publication and selective reporting. The authors have therefore done a service to science in ensuring this study, which does not demonstrate an effect, has been published. They also appear to have adhered to the protocol and not sought secondary outcomes or subgroup analyses that risk generating spurious results.

 3.     It is a fascinating also because the authors were able to evaluate the effect of PZ and treating the infection in people with HIV that was not suppressed by ARVs. This was because it was conducted prior to the WHO recommendation to treat all people living with HIV with ARVs irrespective of CD counts. So this study is “as good as it will ever get” in testing whether treatment has any influence on HIV progression.

 4.     The study is well-written. The background explains studies to date and the gap in the literature leading to this study. Whilst this is a basic expectation of the background section, so many authors do not do this or do it badly, surprisingly; I will use this as an example of good practice in our teaching!  and the methods are clearly explained. The follow up is good and the results well presented.

 5.     It is appropriately reported without any attempt to overinterpret the results. Given the complexity of the study the authors have good numbers recruited. Very few people were started on ARVs during the course of the study and the sensitivity analysis showed this did not, by chance, influence the results, which might have happened if there was chance imbalance in the numbers treated in comparator groups.

 6.     The one point that would really help understand the context-and this is a broader concern with these studies of this kind around treating helminths in HIV (including the studies of albendazole used for soil transmitted helminth infection in people with HIV) is the ambiguity around the purpose of the study: is it truly believed by the authors that this could be potentially important component of treatment by delaying progression of the disease as stated in the first sentence of the discussion? Or that treatment of schisto could have some public health impact on transmission as outlined at the end of paragraph 3 in the introduction? Or is it simply a randomised explanatory trial to elucidate immune mechanisms with helminth infection? This is the only strongly recommended change I would want to see in the amended version.

***Response: Much as we are fascinated by the immunomodulating effects of helminths, and the wider implications of this for human health, we confirm that our intention in conducting this relatively large trial was to obtain conclusive information (to the extent possible) on whether treating schistosomiasis has significant benefit for controlling HIV replication and progression.  Therefore we believe that the statements in the introduction reflect our true position prior to conducting the trial.***

 7.     With the absence of any demonstrable effect in this study, it seems most unlikely, given the dramatic effectiveness of ARVs in viral suppression, that further studies would be worthwhile. Indeed, given patients would need to be on ARVS, with such small putative possible effects of the treatment, the studies would have to be extremely large, probably so large that no-one would fund them. I think it would be extremely worthwhile reporting in their discussion. 

***Response: This is a thought provoking comment.  We agree that it is not likely to be worth testing the effects of treatment of S. mansoni on HIV progression any further.  However, although antiretroviral treatment is recommended on a “test and treat” basis in high HIV transmission settings, such as fishing communities, this is not easy to implement. So complementary interventions which may be easier to implement would be worth consideration.***

# Peer review: Reviewer response for version 1

**DOI:** 10.21956/wellcomeopenres.15987.r33493

**Published:** 2018-07-26

**Authors:** Judd L. Walson

**Affiliations:** 1 Departments of Global Health, Medicine (Infectious Disease), Pediatrics and Epidemiology, University of Washington, Seattle, WA, USA

This is an interesting manuscript presenting the results of a randomized trial comparing high-intensity treatment (frequent quarterly treatment) vs low intensity (annual) of schistosomiasis in HIV co-infected individuals. This paper adds to a previous RCT demonstrating lack of benefit with the use of empiric quarterly praziquantel in HIV infected individuals ^1^. Overall, this was an ambitious attempt to conduct a rigorous trial given considerable challenges in the selection of appropriate patients, potential for alternative treatments (such as ART) to impact the outcomes and need for high retention.

 Unfortunately, there are a number of critical issues with the design and conduct of the trial that make the interpretation of these data difficult.

1. Overall, one cannot expect to see differences in HIV VL above and beyond reductions that would be expected with initiation of ART in antiretroviral naïve individuals. As such, the actual population for whom any expected benefit would need to exclude these individuals (which was done in a secondary analysis). Including these individuals in the primary analysis is problematic as they are not going to benefit from the intervention and they only serve to dilute the impact amongst the population who may benefit. Per my calculations, removing these individuals would leave 81 individuals per arm who were treated and did NOT receive ART. This suggests that the actual study was dramatically underpowered to detect the effect size for which it was designed. This is a significant limitation of the study. In addition, the fact that 12 individuals had undetectable VL at baseline is concerning, suggesting either that there were individuals who were included that had already initiated ART or that there were some fundamental issues with the laboratory in detecting VL. While it would be expected that some individuals in a population could be long-term non-progressors and have low to undetectable VL in the absence of treatment, the number observed here is quite high and raises some concerns.
2. The inclusion of a group of HIV infected, schisto negative “controls” does not add to the study. These individuals are likely to be different in many unmeasured ways from the coinfected population and any comparison of this group is likely subject to considerable confounding. I found that the inclusion of this group detracted from the quality of the overall study.
3. The very high rates of LTFU in this study are concerning. Other RCTs with a similar length of follow up in East Africa among HIV infected adults achieve retention rates of greater than 90-95%. The high rates of LTFU suggest that there may have been differential loss and have introduced significant bias.
4. Important to note that the intervention also included albendazole – any observed effect could also have been attributed to this.
5. The exclusion of individuals with high intensity infection, while perhaps ethically necessary, is problematic for the interpretation of these data. It is likely that these are the individuals most likely to benefit from the intervention and the exclusion of this group makes it difficult to draw any conclusions from these results.
6. Please explain how allocation concealment was maintained. It appears that study staff were provided the randomization lists and allowed to sequentially assign groups. This is subject to bias and may not have resulted in true random allocation. If this was indeed the case, please restate that this was a pseudorandomized trial.
7. Please explain what the authors mean when they say that in each group one participant was “randomized in error”.
8. In table 2, please clarify the CD4 count values presented.
9. The authors suggest that there was previously only one RCT in Zimbabwe evaluating HIV outcomes. This is not the case (as noted above). Please review the literature to ensure that all relevant prior trials are summarized and included in the discussion.

I have read this submission. I believe that I have an appropriate level of expertise to confirm that it is of an acceptable scientific standard, however I have significant reservations, as outlined above.

## References

[1] WalsonJSingaBSangaréLNaulikhaJPiperBRichardsonBOtienoPMbogoLBerkleyJJohn-StewartG: Empiric deworming to delay HIV disease progression in adults with HIV who are ineligible for initiation of antiretroviral treatment (the HEAT study): a multi-site, randomised trial. *The Lancet Infectious Diseases*.2012;12(12) : 10.1016/S1473-3099(12)70207-4 925-932 10.1016/S1473-3099(12)70207-4 22971323

# Response

**Published:** 2019-03-26

**Authors:** Andrew Abaasa

**Affiliations:** MRC/UVRI and LSHTM Uganda Research Unit, Uganda

**Effect of high-intensity versus low-intensity praziquantel treatment on HIV disease progression in HIV and *Schistosoma mansoni *co-infected patients: a randomised controlled trial**

**Abaasa et al**

**Response to the reviewer’s comments**

**Reviewer One**

**Judd L. Walson**, Departments of Global Health, Medicine (Infectious Disease), Pediatrics and Epidemiology, University of Washington, Seattle, WA, USA 

 Approved with Reservations

 This is an interesting manuscript presenting the results of a randomized trial comparing high-intensity treatment (frequent quarterly treatment) vs low intensity (annual) of schistosomiasis in HIV co-infected individuals. This paper adds to a previous RCT demonstrating lack of benefit with the use of empiric quarterly praziquantel in HIV infected individuals ^1^. Overall, this was an ambitious attempt to conduct a rigorous trial given considerable challenges in the selection of appropriate patients, potential for alternative treatments (such as ART) to impact the outcomes and need for high retention.

 Unfortunately, there are a number of critical issues with the design and conduct of the trial that make the interpretation of these data difficult.

 1.     Overall, one cannot expect to see differences in HIV VL above and beyond reductions that would be expected with initiation of ART in antiretroviral naïve individuals. As such, the actual population for whom any expected benefit would need to exclude these individuals (which was done in a secondary analysis). Including these individuals in the primary analysis is problematic as they are not going to benefit from the intervention and they only serve to dilute the impact amongst the population who may benefit. Per my calculations, removing these individuals would leave 81 individuals per arm who were treated and did NOT receive ART. This suggests that the actual study was dramatically underpowered to detect the effect size for which it was designed. This is a significant limitation of the study. In addition, the fact that 12 individuals had undetectable VL at baseline is concerning, suggesting either that there were individuals who were included that had already initiated ART or that there were some fundamental issues with the laboratory in detecting VL. While it would be expected that some individuals in a population could be long-term non-progressors and have low to undetectable VL in the absence of treatment, the number observed here is quite high and raises some concerns.

***Response: This is a good observation, however, it may not invalidate the trial findings because***

***i)               When you exclude from the analysis participants that initiated ART during follow up, which we did in the sensitivity analysis, given that the numbers come to 81, the study power becomes 84.4%, which is below the assumed 89% at the trial onset but above the conventional 80%.  With 84.4% power, and under similar assumptions stated in the sample size estimation in the paper, we would still expect to demonstrate a difference if it truly existed.***

******

***ii)              Indeed the 12 individuals were long-term non-progressors and had low or undetectable VL throughout follow up. We retested them for HIV sero-status to confirm they were truly positive and revisited them to make sure they were not on ART treatment.  It is unlikely that the laboratory had issues in detecting VL because the undetectable VL was consistent for the 12 participants. Furthermore, the median and interquartile VL for participants that started ART was 51474 copies (IQR=16361 – 151520) before starting ART and reduced to 66 copies (IQR 10 - 371) at their last assessment after ART initiation.***

 2.     The inclusion of a group of HIV infected, schisto negative “controls” does not add to the study. These individuals are likely to be different in many unmeasured ways from the coinfected population and any comparison of this group is likely subject to considerable confounding. I found that the inclusion of this group detracted from the quality of the overall study.

***Response: It is plausible that an infection such as schistosomiasis exacerbates HIV progression and that the effect is not reversible by treatment - because of persistence of helminth antigen, for example, and lasting impact on the immune system.  As indicated in the discussion, we consider the results from the comparison group useful to address this possibility – there was no substantial difference in progression between the comparison group and either treatment arm.  We do agree, however, that there were important (and largely inevitable) differences between the comparison group and the infected groups and we acknowledge that all comparisons with that group should be taken with caution and as secondary results. We draw our major findings from the two randomized arms.***

 3.     The very high rates of LTFU in this study are concerning. Other RCTs with a similar length of follow up in East Africa among HIV infected adults achieve retention rates of greater than 90-95%. The high rates of LTFU suggest that there may have been differential loss and have introduced significant bias.

***Response: The retention of 96 (85.9%) in the high intensity, 97 (85.8%) in the standard treatment arms and 107 (78.1%) in the comparison group at 60 weeks were greater than the average 75% reported in longitudinal studies in the fishing communities (a population which is highly mobile due to fluctuations in the fishing seasons). In fact, at the study onset, we assumed a loss to follow up of 20% based on the studies in the fishing communities. The reference is indicated in the sample size estimation section in the methods. It is true that other non-fishing populations have better retention rates because they tend to be more stable than the fishing population. As we report, the number of participants lost to follow up was similar between the trial arms 15.1% in the high intensity and 14.2% in the standard group. Both below the assumed 20% at the trial design stage.***

 4.     Important to note that the intervention also included albendazole – any observed effect could also have been attributed to this.

***Response: In our background, we describe previous results on effects of albendazole.  However, none was given in this study until after the primary outcome measurement at 12 weeks, Thereafter it was given to all groups at the same time points and therefore would be unlikely to influence the comparisons made.***

 5.     The exclusion of individuals with high intensity infection, while perhaps ethically necessary, is problematic for the interpretation of these data. It is likely that these are the individuals most likely to benefit from the intervention and the exclusion of this group makes it difficult to draw any conclusions from these results.

***Response: It is good the reviewer acknowledges that participants with high worm load benefited from immediate treatment. Indeed this was done for ethical reasons. We acknowledge that the results of this paper may not directly be applicable to HIV Schistosoma co-infected patients with egg count >2000 eggs/g. Of note, however, this was the reason for exclusion for only 23 participants, so a minority group.***

 6.     Please explain how allocation concealment was maintained. It appears that study staff were provided the randomization lists and allowed to sequentially assign groups. This is subject to bias and may not have resulted in true random allocation. If this was indeed the case, please restate that this was a pseudorandomized trial.

***Response: This is a good observation. We have edited the manuscript to reflect the randomisation process as it happened. Allocation concealment only happened at the point of treatment allocation. Treatment was not concealed since it was directly observed, and with varying schedules.***

******

***We have therefore included this statement in the manuscript to improve clarity.***

******

***The study statistician generated two randomisation lists: i) containing trial numbers to which eligible participants would be allocated the next available number sequentially ii) containing trial numbers and the allocation arms. List (i) was held at the trial clinic, while list (ii) was held by the trial statistician. When an eligible participant was recruited on the next available number on the randomisation list (i), the statistician was contacted for the treatment allocation on list (ii).  *** 

 7.     Please explain what the authors mean when they say that in each group one participant was “randomized in error”.

***Response:  As a quality control measure, a clinician that did not participate in the assessment of eligibility and randomisation exercise revisited the eligibility procedures for each participant before treatment was given. Two ineligible participants were identified by this control measure and excluded from the trial before being treated, although they had been allocated a trial number. Coincidently, the two participants would have been allocated to the different randomisation arms. *** 

 8.     In table 2, please clarify the CD4 count values presented.

***Response: The VL were transformed to log base 10 while the CD4 were transformed on the natural logarithm. These have been clarified in the Table 2’s footnote.***

 9.     The authors suggest that there was previously only one RCT in Zimbabwe evaluating HIV outcomes. This is not the case (as noted above). Please review the literature to ensure that all relevant prior trials are summarized and included in the discussion.

***Response: We focused on the RCT in Zimbabwe because it was the only previous trial to examine effects of treatment among individuals of confirmed Schistosoma infection status.  We have clarified this in the discussion.    We have also added reference to Dr Walson’s HEAT trial in the introduction, and apologise for our earlier omission of this.***
